# We need to rethink production technology for meat-packers—the old cutting table is being revived

**DOI:** 10.1093/af/vfab077

**Published:** 2022-04-30

**Authors:** Lars L Hinrichsen, Haiyan Wu, Kristian D Gregersen

**Affiliations:** Danish Technological Institute, DMRI, Taastrup, Denmark

**Keywords:** artificial intelligence, capacity utilization, meat processing, productivity, robotic automation, work environment

**Implications**

• Traditional production lines will become much less abundant, and robotic production cells will conquer the arena propelled by low cost and advanced sensors and artificial intelligence.

• A leap step is taken toward a production system operating 24 h 7 d a week with the first robotic production cell fabrication underway at a Danish meat-packing facility. Complex operations will be undertaken by a set of self-learning collaborating robots.

• Knowledge and self-learning algorithms will become an important asset on the meat-packers balance sheet. Existing technology will be disrupted, and first movers will get ahead of their competition: The moment for decision is coming close.

## How We Ended Up Here 

The wonderful story about Mr. Henry Ford visiting a Chicago slaughterhouse almost a hundred years ago is truly enjoyable. A meat-packers disassembly line inspired Ford Motor Company to organize its production in continuous assembly lines and sparked the industrial revolution further into higher efficiencies and fewer production flaws. The story makes people in the meat industry proud, and a visit to a modern slaughterhouse clearly demonstrates how far efficiency and productivity can be driven with sophisticated management systems, skilled employees, and highly specialized automation technology.

Of course, there are big differences among the various species of animals that are being processed. In general, the level of automation is higher, the smaller the carcasses are. Poultry processors are usually highly automated, whereas beef processors are much less automated. The same observation goes for the line speed, where poultry processors can handle 20,000 carcasses per hour, pig processors 750 per hour, and beef processors 300 per hour.

**Figure 1.**
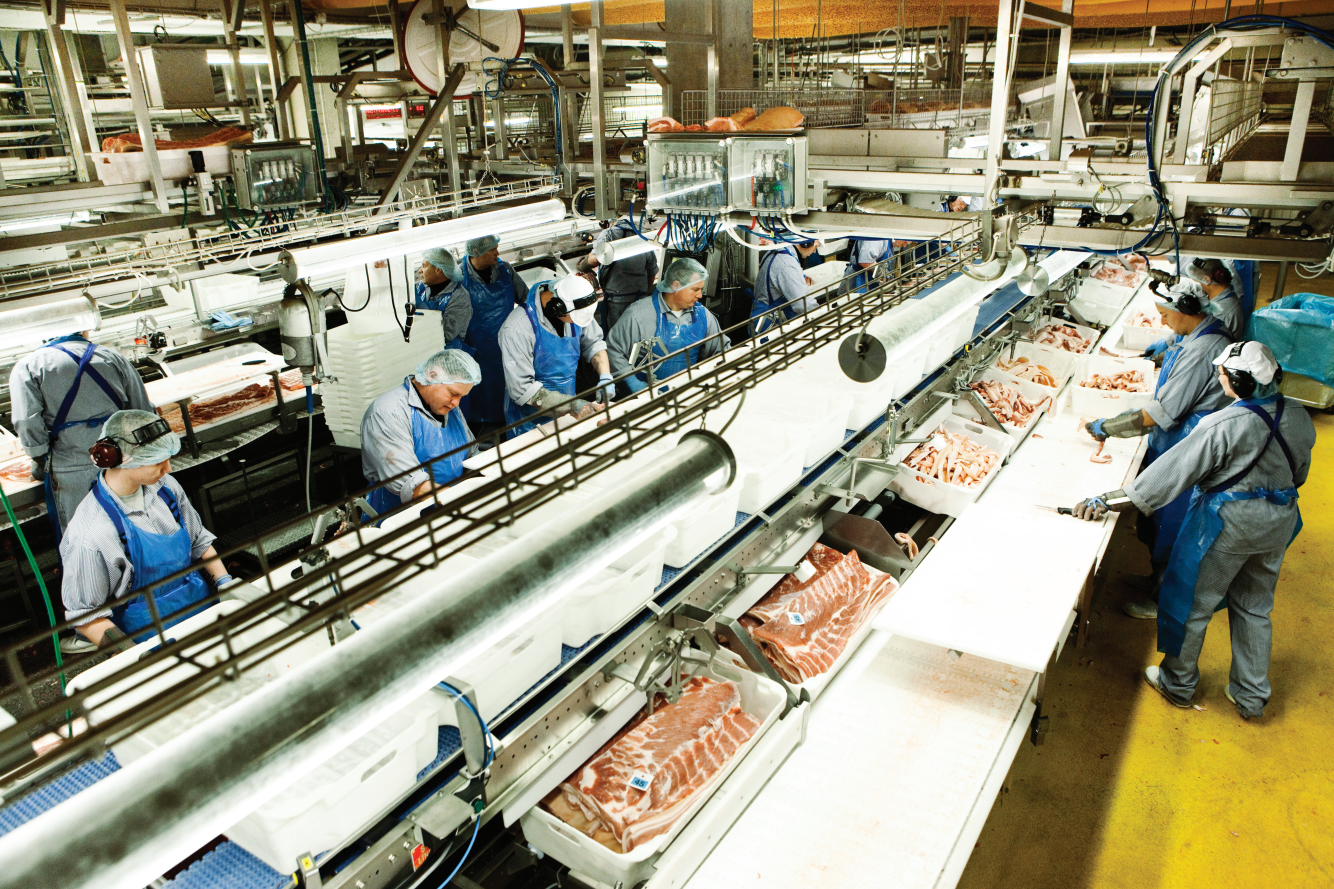
Traditional line production in a pig slaughterhouse.

But the classical line production (see Figure 1) that we know from the meat industry is challenged, and there are several reasons for that. First of all, there is an upper limit to how fast a production line can run. To some extent, this is compensated by designing longer production lines with more people. If there is access to low-cost workers, it may work. But at a certain point, it will become unproportionally more costly to include more workers at the production line, because the consequence is longer production lines, which will increase costs per unit. Moreover, countries with access to low-cost workers are observing increasing salary costs and their particular production setup may risk becoming inefficient which drives the cost of production up.

Another reason is that it is becoming increasingly difficult to attract workers to the meat industry. The work environment is strenuous and often the salary is low. Other sectors offer more attractive jobs, leaving the meat industry with a big recruitment problem.

The supporting automation technology is yet another reason why we may see the end of line production in the meat industry (Barbut, 2020). Because line speed is very high in the slaughterhouses, the automation technology must be specialized and adapted to the extreme production conditions. That is costly and with a limited market size, the incentive to innovate may be too low. Moreover, the technology must be sufficiently robust for frequent sanitation which adds to the production costs. 

On a more theoretical note, it can also be observed that the utilization of the automation technology is low and may be only 30% of the full capacity. This is the nature of line production due to the design. The automation technology is fitted to the production line and is awaiting the carcass to approach before the automation technology can perform the processing. Often the automation technology is standing idle a lot more than the processing time, and from an engineering point of view, this is highly inefficient and adds to production costs.

When a production line is running at full speed, it has a high productivity, but experience shows that it is not uncommon that a single incident along the line causes line stops frequently. An uptime less than 80% is not uncommon and sometimes even as low as 50%. This clearly demonstrates the vulnerability of classical line production and the extreme demand for uptime of the automation technology fitted to the line. Keep in mind that any modern slaughterhouse receives animals on an ongoing basis and that breakdown can be detrimental to the inflow of animals and the preceding logistics. Uptime is king.

So instead of designing the automation technology to fit the production line, why not design the production setup to fit the automation technology? We need to rethink the production design and it may well mean that we will reinstall the old cutting table where many processes were done by one operator in parallel instead of lines. In fact, this is exactly the development that the Computer Numerical Control (CNC) industry went through in the 1990s and that the automotive industry is going through more recently (Angerer et al., 2015)—migrating from classical line production into what we will call cell-based production. Many operations are performed in a cell with many cells in parallel contrary to the line production where one operation is performed at a time. A cell-based production in the meat industry compares to a very sophisticated cutting table.

## What Is Cell-Based Production

**Figure 2.**
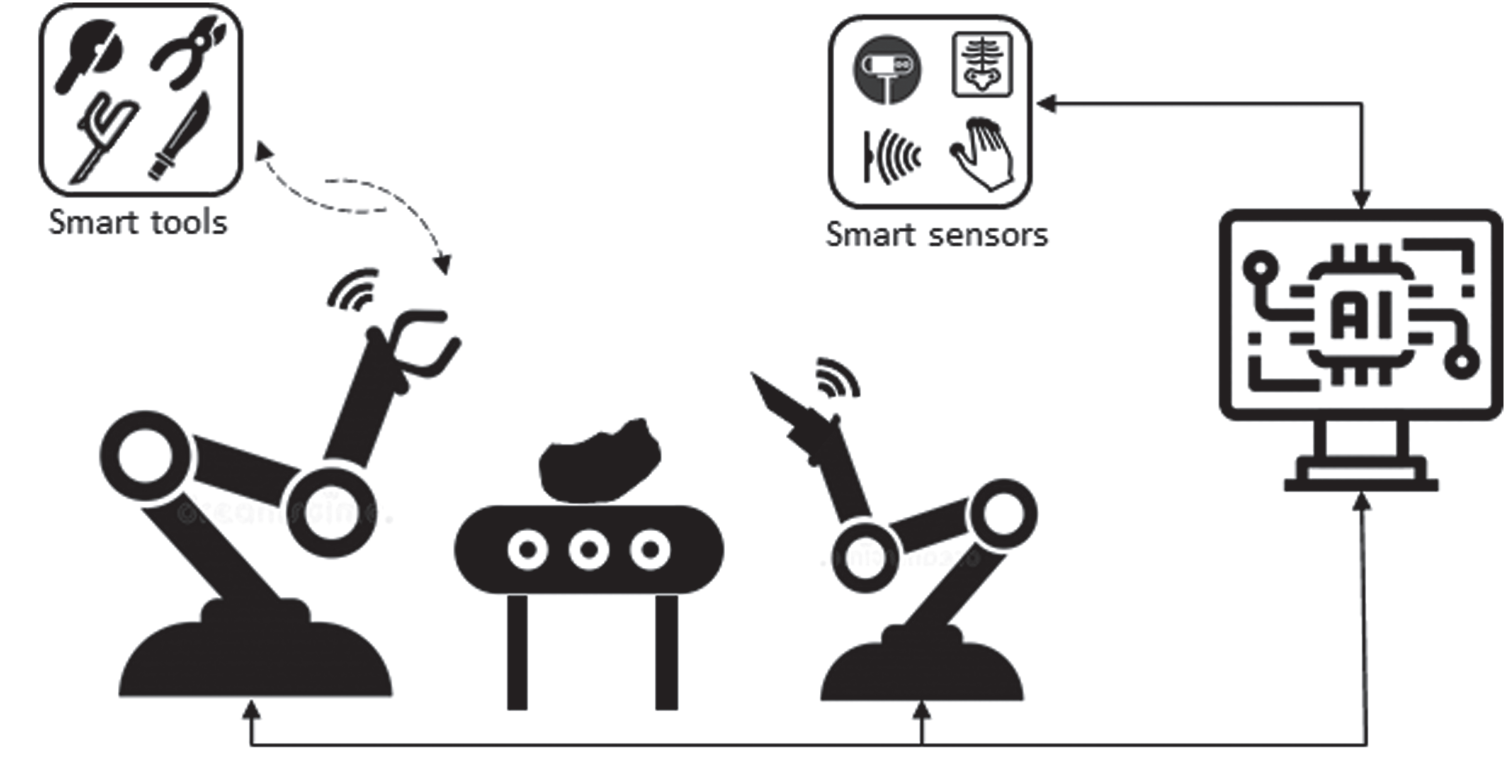
An example of cell-based production equipped with multiple robots, smart tools, smart sensors, and artificial intelligence (AI)-based intelligent control system.

Ideally, a production cell is a delimited area where an item is being processed by numerous operations. It can be done by people, but in this context, it is the application of robots that makes it relevant. A robotic production cell can consist of one or more robots that process the carcass with many operations (see Figure 2).

The robots will work simultaneously or even collaborate and ideally process an entire carcass including cutting and deboning. A robotic production cell is particularly relevant for larger carcasses due to the labor intensity and at this point lack of cost-efficient available automation technologies on the market.

Why are robotic production cells interesting? Part of the answer lies in the fact that it enables much more powerful innovation. Until now, the meat industry and their technology providers have developed costly specialized automation technology, but the application of standardized industrial robots opens the door into the huge innovation storm that takes place in assembling industries (Brogårdh, 2007). The automation technology can to a much larger extent rely on standardized industrial solutions at a much lower cost. Simply said, the technology providers to the meat industry will not anymore need to engineer complicated mechanized solutions but simply include best practice robots and instead focus on tools and control algorithms. The powerful robot innovation storm will have a natural inflow into the meat industry as well.

In the pig industry, kill lines are already automated, and it is likely that robotic production cells will be a next-generation choice for technology providers. But a little further downstream, it has been almost impossible to automate the cutting and deboning areas. The product mix is high, production series may be short, and cutting operations are complex. In this situation, the robotic production cell is highly relevant and probably a feasible way to improve efficiency in this area.

Robotic production cells will be organized in parallel to match the needed capacity of the slaughterhouse; basically, redundant robotic production cells that can perform the exact same operations. This redundancy implies that the risk of total production stops is much lower compared with the classical line production. If one production cell breaks down, the remaining production cells can still function, and production will continue although with a lower capacity. This is a huge advantage compared with the present situation.

## It’s All about the Algorithms

Establishing a robotic production cell is a big investment to any meat processor or technology provider. But the explosive development of technology creates a lot of tailwind. Sensors are key to the operation of the robots, and obviously, machine vision is an integral part of the function. The development is rapid, and cameras are becoming cheaper and more powerful year by year. Especially, three-dimensional-,Time-of-Flight-, and multispectral cameras are useful to give input to the robot control system in combination with tactile feedback systems (Kumar, 2018).

However, clever design of the tools mounted at the end-effector of the robots will make the entire operation much simpler. The correct choice of materials, design, and operation of the tools is the key to get optimal yields and correct product quality. Although tools are being designed as generic as possible to make sure that they can be used for several operations, they are still specialized to some extent. Therefore, more robots carrying different tools are needed. However, more robots are eating square meters, and there are never sufficient square meters available at the slaughterhouse. Automatic shift of tools is much preferred and will be a way to keep the footprint down of the robotic production cell.

In the end, vast amounts of data are being created, and the control algorithms must be able to handle them sufficiently fast to be able to control the robot. Developing algorithms will be the true shift of paradigm, because the algorithms will not only be hard coded for a number of operations, but it will also be self-learning and adaptive. Once in operation, the algorithm will be improved based on the ongoing production. It can learn gradually about how to cut to other specifications, and it can accommodate the biological variation among each carcass, meat cut, or in longer terms, species change over time.

The implications of this are huge because this is exactly what is needed to handle small production series and a complex product mix. Ideally, the robotic production cell can operate with an individual cutting pattern optimized for each individual item—a true hyperflexible production.

As algorithms are self-learning, they will eventually become adapted to the products and procedures of the slaughterhouse. They will become part of the critical knowledge base that any company tries to protect and that is often part of the company’s competitive edge. Moreover, the algorithms represent a powerful way to transform tacit knowledge into solid documentation. The algorithms can be duplicated and proliferated, and in an international production setup, this is a powerful way to create an aligned and high-quality production, where trained algorithms can be introduced into new robotic production cells anywhere in the world. Furthermore, the algorithms can be interconnected across robotic production cells, across production plants, and across borders—what a powerful learning system!. Just like the roots of a tree. In a future not so far away, the algorithms may be the most important asset on the balance sheet.

## World’s First Robotic Production Cell for Pig Slaughterhouse Fabrication Is Underway

**Figure 3.**
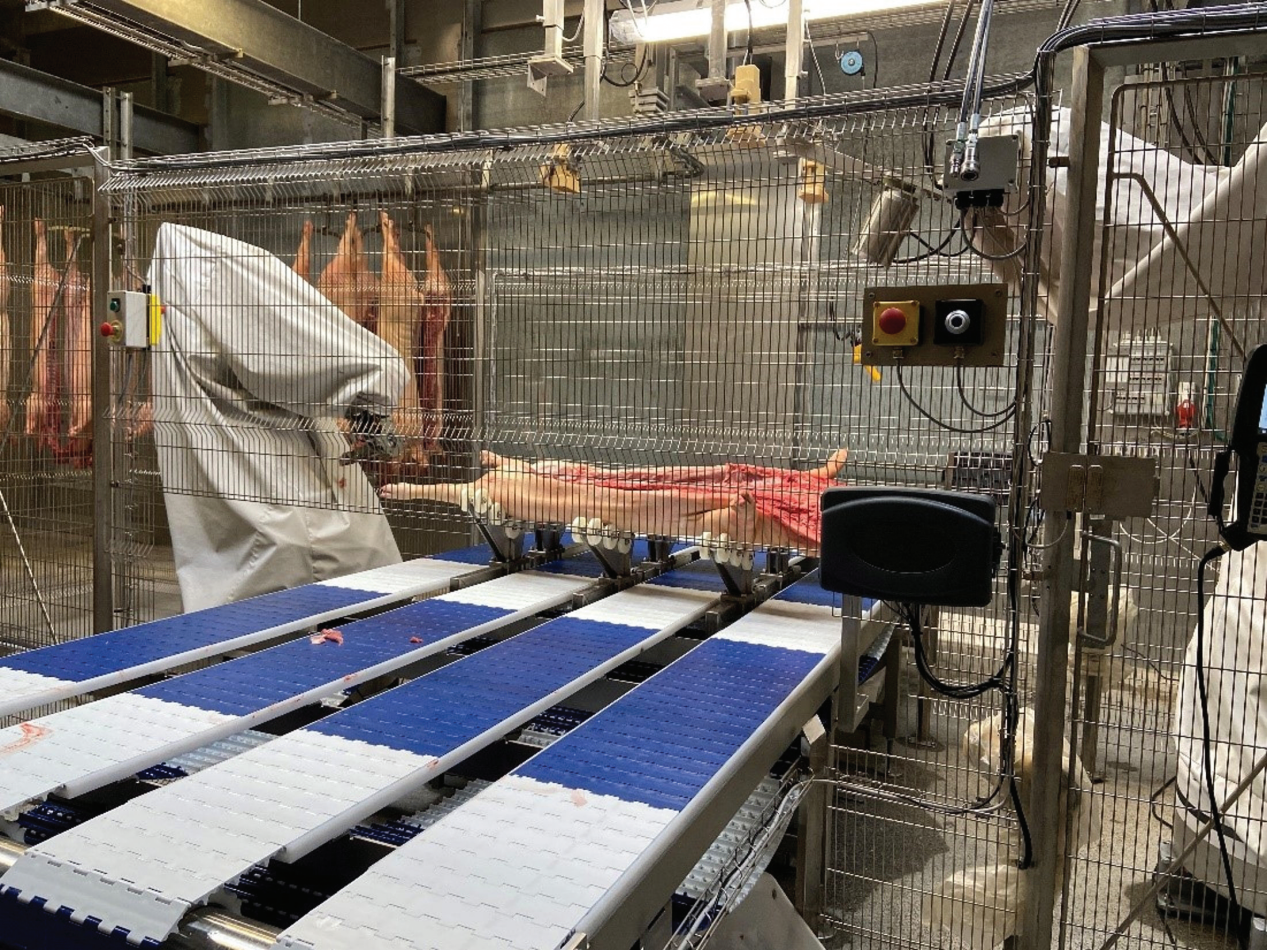
A prototype of multifunctional robotic production cell under development.

The concept of robotic production cells may hold the key to a new cell-based production paradigm at the slaughterhouse. However, to turn the idea into reality requires acting. To this end, the development of the world’s first robotic cell for pig production (see Figure 3) at a Danish slaughterhouse has started with the aim to investigate the practical implications in a realistic setup (Philipsen and Moeslund, 2020; Wu, 2020).

As cell-based production completely disrupts the traditional line production, it is very challenging to integrate the robotic production cell into existing facilities. If it was possible to introduce cell-based production across the entire slaughterhouse at the same time, these challenges would of course not exist. However, the exploration of robotic production cells has only just begun, and a factory-wide installation is still very far away. A realistic path toward cell-based production will identify and replace parts of the traditional production line, and, therefore, solving problems related to integration is crucial. For this reason, the layout of the robotic production cell as well as the choice of products to be processed and how these will flow in and out have been considered. Six operations have been identified, all of which are carried out at the same location after the cold carcasses leave the chilling area and enter the primal cutting. The six processes are toe cutting, tenderloin extraction, ear cutting, stab wound removal, head cutting, and trisection. These choices make it feasible to install the robotic production cell in the existing production flow at the slaughterhouse.

For the tests carried out, the carcasses are brought directly from the production and arrive in the test facilities hanging on rails from the ceiling. The carcasses are taken down onto a conveyor that carries the carcasses into the robotic production cell. After the carcass arrives inside the robotic production cell, it is analyzed with sensors and cameras to extract relevant information. The six operations are then executed by several robotic arms in parallel using dedicated tools developed for each individual task. Everything is orchestrated by a software control system running on a powerful PC next to the robotic production cell. The software is completely modular with key components running on separate threads in parallel to take full advantage of the available computing power. The software structure provides maximal flexibility and makes it easy to be extended with new modules, for instance for additional algorithms, sensors, cameras, tools, or robots.

The core idea is to transfer the complexity from dedicated mechanical design to an intelligent software control system. This strategy keeps the cost down on custom machinery and adds the flexibility to reconfigure the system on the fly. It also drastically reduces the development time and facilitates the exploration of more innovative solutions for highly complex manual operations. The ongoing development at the test facilities is focused on intelligent control systems and smart tooling, including a generic fast tool change.

One of the main challenges is designing the intelligent part of the control system. The question boils down to how the relevant information is extracted from various sensor and camera inputs to guide the robots. The progress in research and development in artificial intelligence, in particular deep neural networks (LeCun et al., 2015), has made it feasible to introduce these algorithms in the automation solutions at the slaughterhouses.

Designing robust deep learning algorithms has always been difficult regardless of the application domain. However, the slaughterhouse takes the usual challenges to a new level due to the large variations in the input and the strict requirements on precision and capacity. Experienced butchers are both skilled and fast, and the individual processes are already highly optimized in the existing line production setup. Consequently, the algorithms must be very fast and extremely precise to make the robotic production cell a feasible replacement. The large biological variation and variations introduced from upstream processing by human operators or machines make this task extremely challenging.

**Figure 4.**
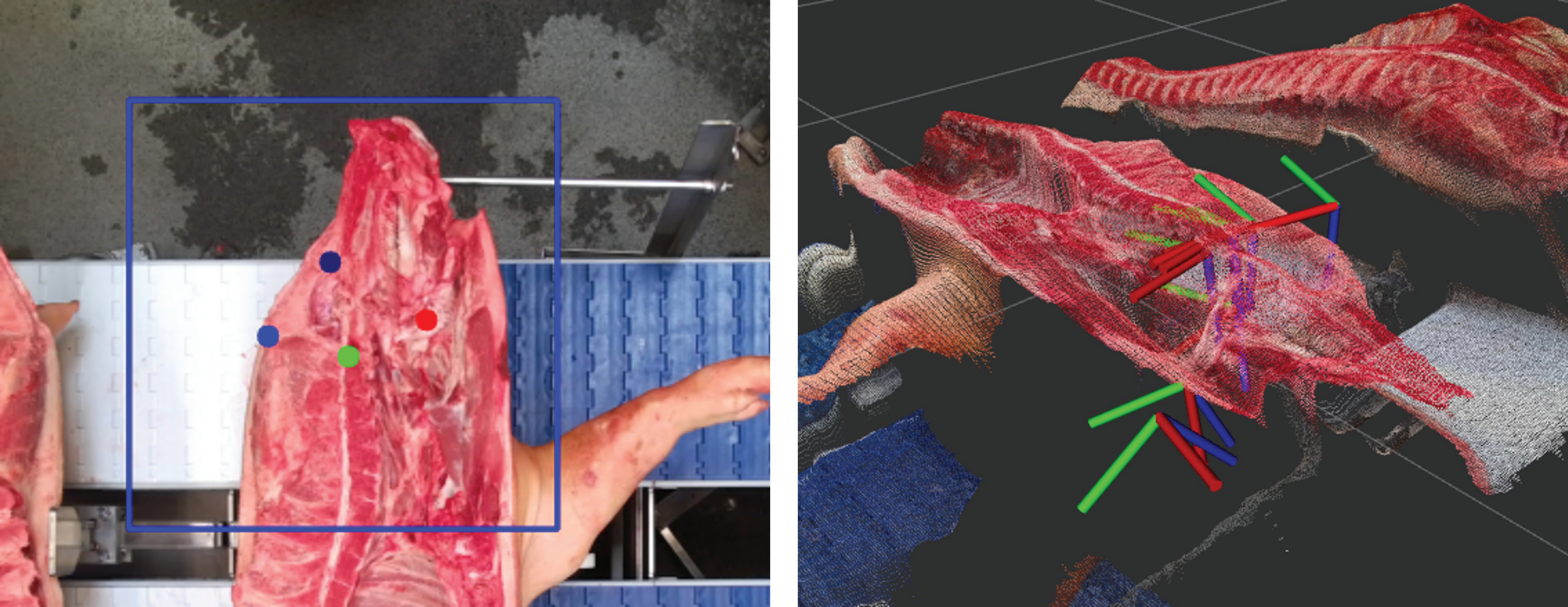
Landmarks detection and cutting path generation for head cut in robotic production cell.

In the robotic production cell, the algorithms are provided with images of the carcass recorded by several three-dimensional cameras located inside the cell. During initial tests, it was quickly realized that common off-the-shelf deep learning algorithms, for instance, based on semantic segmentation (Lateef and Ruicheck, 2018) or object detection (Liu et al., 2020) have difficulties meeting the requirements. To resolve these issues, a custom neural network architecture is embedded in the core of the control system. The design relies on many discoveries made during intense tests in the robotic production cell concerning all parts of the pipeline, from data collection and annotation strategies, network architectures, training methodologies, tool design, and cutting strategies. The resulting algorithms (see example in Figure 4) are robust, precise, and easily run in real time, adapting the cutting patterns to each individual carcass for all six operations under investigation.

Although several milestones have already been reached, many challenges still lie ahead. The robotic production cell test facility is a first attempt to bring the abstract concepts of multifunctional cell-based production into practice for the meat industry. The task is immense and, even when broken down into parts and solved one at the time, uncharted territory is explored for every step taken.

So, what is next? Sometimes it is forgotten that intelligent control systems only provide intelligent solutions at the moment of creation. Automation solutions degrade over time because developers cannot predict what will happen in the future. Will the carcasses increase in size? Or maybe new machinery upstream introduces new physical features on the carcasses? Preparing for the next step, the potential application of a range of online learning algorithms is currently under investigation, for instance, reinforcement learning (Francois-Lavet et al., 2018), which exactly addresses this problem. In fact, these technologies turn the situation upside–down. From the time the intelligent control system is installed, the quality will not degrade but instead improve. This happens through a feedback loop that analyzes the quality of the operations and based on this allows the system to self-optimize while running. The successful implementation will unleash some of the latest developments in artificial intelligence at the slaughterhouse.

## Technology Trends

The digital development is so fast that even just understanding the new possibilities is a challenge. Everything becomes cheaper and Moore’s law (Schaller, 1997) is also applicable in this situation. Risk for the slaughterhouse is obvious that once the capital expenditures are decided, the company is stuck with the decision, and tapping into new technology may lead to a loss of the investment. But the production paradigm with robotic production cells is a bit different because a large part of the technology is modularized and based on standard platforms. It compares to consumer electronics where we are used to frequent software updates for that will improve performance or add new features this is also the case for robotic production cells. The Tesla vehicle is a brilliant example of a platform that can be remotely updated, and extra performance can be purchased as downloads (Chen and Perez, 2018). In the near future robotic production cells will recieve frequent software updates that will improve performance and prolong the lifecycle of the investment. 

But there is an even more important aspect especially when it comes to capital expenditures and hardware. Hardware updates, new tools, new functionalities, new sensors, and so forth can be retrofitted in the cells. With the natural redundancy in a setup with robotic production cells, the actual retrofitting can be done simultaneously with production, and no stops are needed. A robotic production cell should not be looked upon as a complete machine but rather a versatile platform that continuously can be improved and adapted to new conditions.

## The Business of Biological Variation

Most slaughterhouses are sorting carcasses based on value, size, and other parameters. There are millions of dollars hidden in correct sorting of the carcasses, which is basically to match carcass composition with customer specifications and expectations. Modern slaughterhouses have big cooling facilities to handle carcass variability. The reason is that the workers manually must cut to a certain specification based on the type of carcass that is being presented to them, and the only way to do that is to sort the carcasses in a cooling facility before fabrication.

But that complexity disappears with the introduction of robotic production cells. In the ideal world, sorting is no longer needed because the production cell can cut the carcass optimally based on the biological variation. It assumes that there are sufficient appropriate customer orders received, but overall, it is a big simplification of the slaughterhouse logistics. A much simpler design is needed and energy for cooling can even be saved.

As a bonus, it will be possible to introduce complete traceability down to each cut leaving the robotic production cell; a challenge that remains unsolved today and is currently handled with batch traceability in most cases.

## There Are Still Some Steps to Take

It has been demonstrated that it is possible to make a robotic production cell for a pig slaughterhouse. Technologies are available and the business case is very attractive. But of course, there is still a lot of work ahead.

Technically there are three main areas that must be solved. First of all, it is necessary to develop a very fast shift of tools may be even on the fly. Robotic production cells will at least in the beginning need to be fitted into existing facilities, so a small footprint is essential. Shift of tools is common in other industries that use robots, but the challenge is the speed.

Secondly, a sanitation system must be developed. Probably the most important thing with robotic production cells is that they enable production 24/7. The factory can increase its capacity maybe by 30% without adding any cost of production, and this is where the real business case for this production setup is hidden. This will require that the cells can be cleaned in place (CIP). Multiple robotic production cells create a high redundancy in the production system and, one cell can be cleaned, while the remaining cells are producing. There already exist CIP systems for robot solutions, but they have never been tested in slaughterhouses or in this kind of robotic production cells. 

Thirdly, logistics around the robotic production cells must be in place. Depending on how many operations the cell will be able to provide, the logistics around the cell will be complex. It is necessary with a simple conveyor design around the cell to make logistics as smooth as possible preferably without any transport boxes. It is easy to underestimate the risk of complex logistics, and in this case, it is not different.

In the early days of automation, it was popular to state, that we want so much automation, that we can turn off the light in the factory, meaning that no people were necessary for the production. That statement is not relevant anymore, because robotic production cells still need skilled workers around them. Actually, the role of the workers will radically change. Today most of the work is standardized repetitive operations, where each operator is trained in a particular set of operations. With robotic production cells, the role of the operators is not only to oversee a set of cells but also to be the product quality expert. The skilled butcher will become very important, as their skills will be part of the learning input to the algorithms. If the cell meets a situation, where it does not know what to do, the skilled operator will teach the machinery, and the cell will have achieved a new learning input. The job will change from a blue-collar worker to a skilled technician. Jobs will be much fewer, but they will be better paid and less strenuous.

## How to Get There

How can the slaughterhouses get access to this kind of technology? As in all aspects of technology development, someone must be the first mover and probably that is the companies with the most urgent need. But beware that there is a dramatic disruptive potential in this technology. As a robotic production cell is basically a standard industrial platform, it opens for many more technology providers than the industry has today. Previous solutions were highly mechanized and integrated tying the slaughterhouse to the particular technology provider, but with this new production paradigm, the field is completely open, and any robot integrator would potentially be able to enter the arena. Moreover, the slaughterhouses will claim their rights to the algorithms, which will lower the bargaining power of the technology providers. Good news for the slaughterhouses and good news for new to the business technology providers, who wants to enter the arena. 

New technology also means new ways of working. Many slaughterhouses have agreements based on time studies or volumes, and these kinds of agreements will not make any sense with the new technology. As work moves from blue collar to technician type of work, the management approach must change accordingly.

So, the first-mover will not only take the risk but also get ahead of their competition if it is a success and the moment for decision making is coming close.

Self-learning robotic production cells, which are hyperflexible and which can be added with new features, both physically and digitally, represent a disruptive shift in the production paradigm in the meat industry. The circle is closed, and we are back to the cutting table. Maybe, the automotive industry will be inspired again a hundred years after the visit to Swift and Company’s slaughterhouse in Chicago.

## About the Author

**Lars Hinrichsen** is an *executive vice president at Danish Technological Institute, DMRI.* He has a background as a food scientist and holds a PhD in meat technology from the University of Copenhagen and an executive MBA in innovation management from the Technical University of Denmark. He has a strong interest in innovation and automated industrial production and has throughout his career worked with the scientific, technological and practical aspects of the food industry. With more than 25 yr of industrial experience from the international food sector, he has a comprehensive view of what it takes to convert scientific discoveries into business value knowing the industrial demand, the dynamic business environment, and the long-term strategic research in the public sector. He holds various honorary positions in boards and committees, and he is the chairman of the scientific committee in the International Meat Secretariat.

**Haiyan Wu** has a PhD in automation and control from the Technical University of Munich. She has over 15 yr experience in robotic systems design, including adaptive autonomous meat processing, advanced control system with AI-based vision system, high-speed visual servo control, and virtual reality-aided system design. Her current work focuses on intelligent robotic solutions by applying the latest results from the research community to the next generation of slaughterhouse automation.

**Kristian Gregersen** is the production manager at Danish Technological Institute where he is leading the AI development for robotics in the meat industry. He has a PhD in particle physics from the University of Copenhagen and worked for several years as a researcher on the Large Hadron Collider experiment at CERN in Switzerland. He has more than 10 yr of experience in AI, machine learning, and advanced data analysis. His current work focusses on bringing AI to the automation solutions in the meat industry and bridging the gap between the latest developments in advanced algorithm design and the practical implementation at the slaughterhouse.
